# Women on Hospital Boards

**Published:** 1899-12-30

**Authors:** 


### The Institutional Workshop.

### WOMEN ON HOSPITAL BOARDS.

Why is it that women are unrepresented on the boards of so many hospitals? They have made good their claim to a place on school boards, boards of guardians, and parish councils by the admittedly valuable work they have done on them. They are to be found on some hospital boards also, but not by any means in such numbers as we should expect, seeing how obviously useful a field lies open to women in hospital administration.

We have heard only one reason assigned for this, and it is a bad one. At the last election of managers at a large hospital in an important town, two ladies were candidates for election. One gentleman opposed their appointment on the ground that in a hospital the matron was bound to be something of an autocrat, and she might dislike the interference of ladies in her management.

Now, is there any foundation for this notion? Are women better without the supervision of their fellow-women. Let us not deny that lady managers on hospital boards would exercise supervision. That is what they are there for. They would not, in any offensive way, interfere with the matron, and would recognise and admit that she knew more about the management of nurses and patients and of hospital housekeeping than they did; but they would certainly be free to ask a question, and to offer an opinion on the subject. Would this be harmful to the interests of the hospital, which are, after all, the main interests to be considered? We think not. On the contrary, those slanders concerning the treatment of patients in hospitals which occasionally gain currency could be much better suppressed by the explanation that could be given by a lady manager than by all the protests of men, who, however competent they are in conducting the business of the institution, are very rarely conversant with those details concerning which complaints are apt to arise. Moreover, the testimony of a board of management containing women, on such a subject, would be received by the public with much more confidence than would the most eulogistic support given to a matron by a committee containing only men. The lady manager would be the lady visitor of the hospital, who would go through the place with greater authority than can belong to a mere philanthropic visitor, who is permitted to enter more or less on sufferance. She might, in many cases she would, become the trusted friend and sympathiser of the matron, and we think that many matrons would welcome the advent of directors who could understand, better than men could do, the difficulties and responsibilities of her position. To declare that a matron does not want ladies on the board of a hospital is, in effect, to declare that the matron is an unconscientious woman, who thinks she can hoodwink men better than women, and means to do so. If this were the case, it would be so much the greater reason for getting women on the boards of all hospitals. But we are sure that it is not the case, but rather that the reverse is true.

Not in this must we seek the true reason why women are not so numerous on hospital boards as we should wish. It must be sought elsewhere. It may be that, as in so many other things, the rules for the election of managers are so worded that they seem to imply that men only are eligible. Even where the rules do not, strictly speaking, bear this interpretation, if they have for a long time been so interpreted it is difficult to make a change. It can very rarely be advisable for a woman to endeavour to force her way on to a hospital board. But it would be well that, whenever a vacancy occurs, there should be a woman ready to fill it. And this is a point that cannot be left to the last moment. Nothing would do more harm to the prospects of a suitable lady being elected to a hospital board than that suddenly a woman candidate, the one most willing rather than the one most desirable, should be put up for election. At least, in the first instance, the election of the woman candidate should be made sure beforehand. The novelty of the idea should have worn off before the election comes on. As soon as people begin to think of it, it is seen that it is peculiarly fitting that women should be represented on the board of the hospital, but it is just the first effort of thinking that is sometimes wanting. It would be necessary that some of the chief supporters of the hospital should bring it before the minds of the subscribers that there should be a lady among the directors, even before they brought up the name of any particular lady; and then one must be chosen who is known to be interested in the institution—not one who would use her position for the furtherance of some propaganda in which she is still more interested. There is no use on a hospital board or anywhere else for a woman who cannot get on with her male colleagues.

After all, in nine cases out of ten, all that would be needed would be the suggestion that a lady should be added to the board of managers. The common sense of the subscribers would at once answer, "Of course there should." Women have done such good work on school boards and boards of guardians that their fitness for hospital work would never be questioned. But it is necessary that some one should take the initiative. Failing any desire on the part of the existing managers to have a lady among them, the first suggestion might come from the association, which exists in most large towns, to promote the return of women to local boards.

This association is composed of ladies, and surely it would be possible for them, either from among their own numbers or among their acquaintance, to discover women fit, and, after due persuasion, not unwilling to come forward as candidates for a seat on the board of the local hospital. It is to these associations that we must refer the duty of bringing women to take their proper share in the hospital administration.

